# Genetic Association of Mood Swings with Lung Function and Respiratory Diseases

**DOI:** 10.3390/jpm15110550

**Published:** 2025-11-12

**Authors:** Yin Ku, Shasha Li, Dongsheng Wu, Nanzhi Luo, Zhipeng Gong, Binbin Hu, Kejia Zhao

**Affiliations:** 1 Department of Thoracic Surgery, Institute of Thoracic Oncology, West China Hospital, Sichuan University, Chengdu 610041, China; kuyin@wchscu.cn (Y.K.); ssl314@wchscu.cn (S.L.); wudongsheng@stu.scu.edu.cn (D.W.); nanzhiluo@wchscu.cn (N.L.); zp_gong@stu.scu.edu.cn (Z.G.); 2 Department of Radiation Oncology, Cancer Center, West China Hospital, Sichuan University, Chengdu 610065, China

**Keywords:** mood swings, lung function, respiratory diseases, pleiotropic loci, causal relationship

## Abstract

**Background:** Observational studies have linked psychotic disorders to impaired lung function and respiratory diseases, but the potential association between mood swings—a potential predisposing factor for psychotic disorders—and lung health remains poorly understood. **Methods:** Using summary-level data from large-scale genome-wide association studies, we investigated the shared genetic basis and putative causal links between mood swings and lung function, asthma, and chronic obstructive pulmonary disease (COPD). **Results:** Evident genetic correlations in our study were observed between mood swings and FEV1 (*r_g_* = −0.09), FVC (*r_g_* = −0.09), PEF (*r_g_* = −0.09), asthma (*r_g_* = 0.33), and COPD (*r_g_* = 0.28). Local genetic correlation analysis identified 10 significant local genomic regions, including chr17p12-p11.2 and chr16q23.1. Furthermore, cross-trait meta-analysis revealed 55 SNPs shared between mood swings and lung function, 2 SNPs with asthma, and 1 SNP with COPD. A transcriptome-wide association study identified 43 shared genes that largely overlapped with those revealed in the cross-trait meta-analysis, implicating tissues in the nervous, respiratory, digestive, and cardiovascular systems. Mendelian randomization analysis provided evidence that mood swings are significantly linked to reduced FEV1 (OR = 0.85, 95% CI = 0.77–0.93), reduced FVC (OR = 0.86, 95% CI = 0.77–0.96), reduced PEF (OR = 0.82, 95% CI = 0.74–0.91), and an elevated risk of asthma (OR = 2.22, 95% CI = 1.60–3.06) and COPD (OR = 2.02, 95% CI = 1.37–2.98). **Conclusions:** This study highlights a shared genetic basis and putative causal relationship between mood swings and impaired lung function and respiratory diseases, providing genetic evidence that underscores the importance of investigating mood instability in the context of respiratory health.

## 1. Introduction

Mood swings, a prevalent personality trait, is characterized by recurrent, abrupt, and unpredictable shifts in emotional state [1]. Within the UK Biobank, approximately 45% of participants report frequent experiences of emotional instability [2], prompting our focus on the genetic underpinnings of this self-reported trait. Unlike psychiatric disorders, which typically emerge later in life with an episodic presentation, personality traits continuously shape behaviors and biological processes after birth [3]. This persistent effect may significantly affect disease development. For instance, Liu et al. identified that mood swings was causally related to an elevated risk of cardiovascular diseases (CVD) [4]. Zhong et al. highlighted the potential of mood instability to influence autoimmune diseases, suggesting that mindfulness-based interventions could ameliorate related immune dysregulation [5]. However, the potential links between mood swings and both lung function and respiratory diseases remain largely unknown.

Previous research has explored the relationship between psychotic disorders and lung function, as well as respiratory diseases. Park et al. found significantly lower lung function in individuals with depression compared to those without [6]. Based on a large sample of 766,427 in Taiwan, Hsu et al. reported that individuals with bipolar disorder would be more prone to suffer from chronic obstructive pulmonary disease (COPD) when compared with the general public [7]. In a nutshell, these established results highlighted the elevated risk of impaired lung function and a higher possibility of harboring respiratory diseases for individuals with psychotic disorders. Given that mood swings represent a potential predisposing factor for psychotic disorders [8,9], a critical question remains: what are the broader health implications of this trait? This study aims to elucidate the potential causal relationships and shared genetic mechanisms linking mood swings with lung function and respiratory diseases.

By utilizing genetic cross-trait analytic methods alongside the growing availability of high-quality genome-wide association study (GWAS) data [10], researchers can explore the associations between mood swings with lung function and respiratory diseases, while delving into the potential mechanisms. Specifically, genome-wide cross-trait analysis allows for the estimation of genetic correlations, offering valuable insights into underlying etiology and aiding in the identification of causal relationships between traits. Additionally, this approach can identify SNP-level shared signals that elucidate observed genetic correlations, while Mendelian Randomization (MR) is widely utilized to infer causal association [11].

**Figure 1:**
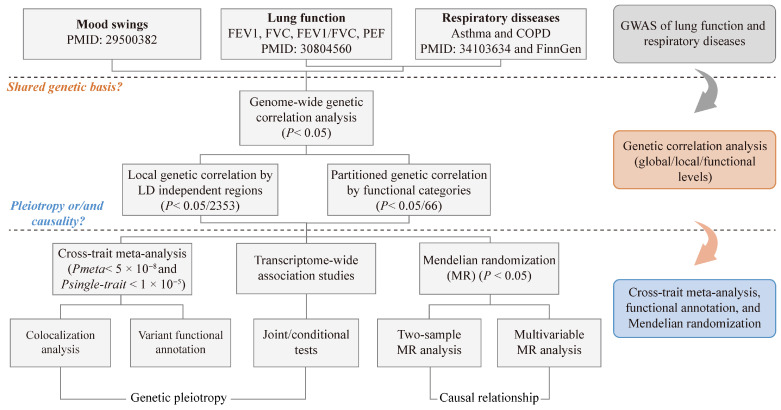
Flowchart of overall study design. FEV1, forced expired volume in 1 s; FVC, forced vital capacity; PEF, peak expiratory flow; COPD, chronic obstructive pulmonary diseases.

This study adopted an innovative genome-wide cross-trait design to explore shared genetic elements and potential causal effects between genetically proxied mood swings (based on a single UK Biobank self-report item) and lung function and respiratory diseases. We initially measured genetic correlations to identify shared genetic elements, followed by a cross-trait meta-analyses to better target pleiotropic loci. To infer potential causal effects, we also considered supplementing two-sample MR analyses, including univariable and multivariable approaches. Figure 1 depicts the systematic conceptual framework of this study.

## 2. Methods

### 2.1. GWAS Summary Statistics

We obtained GWAS summary-level data for mood swings from a GWAS involving 373,733 participants from the UK Biobank [12]. Mood swings were defined by the response to the query, “Does your mood frequently fluctuate?”, which serves as a proxy for genetic liability to self-reported mood instability. Participants who answered “yes” were categorized as cases, accounting for 45%, while the remainder were categorized as controls.

Lung function traits with available updated GWAS datasets contained forced expiratory volume in 1 s (FEV1), forced vital capacity (FVC), FEV1/FVC ratio, as well as peak expiratory flow (PEF), from meta-analyses involving the UK Biobank and SpiroMeta [13]. For asthma, we used GWAS summary data with 352,255 controls and 56,167 cases from the UK Biobank [14]. For COPD, we sourced data with 311,286 controls and 18,266 cases from the FinnGen database (www.finngen.fi/en, accessed on 15 October 2024). Detailed summary statistics are listed in Table S1.

### 2.2. Statistical Analysis

#### 2.2.1. Genetic Correlation Analysis

We first estimated the genetic correlations between mood swings and lung function traits, as well as common respiratory diseases (asthma and COPD), applying linkage disequilibrium score regression (LDSC) implemented in PLINK (v1.9) [15]. This analysis was based on precomputed LD scores derived from well-imputed Hapmap3 SNPs across European populations, encompassing approximately 1.2 million variants [15]. Genetic correlations were estimated on a scale from −1 to 1. To mitigate potential bias from sample 0. overlap between the exposure and outcome datasets, we additionally conducted LDSC using a constrained intercept approach, which enhances robustness in such scenarios [16].

#### 2.2.2. Local Genetic Correlation Analysis

To reveal more signals that exhibit opposing effects at different loci or are specific to certain genomic regions rather than merely emphasize the average estimate across all regions, we calculated local genetic correlations within 2353 predefined LD-independent genomic regions using SUPERGNOVA [17]. Statistical significance was evaluated using the Bonferroni correction, with a threshold set at *p* < 0.05/2353.

#### 2.2.3. Partitioned LDSC Analysis

To determine whether genetic correlations between mood swings and lung function or respiratory diseases were enriched in specific functional regions, we computed annotation-specific genetic correlations using partitioned LDSC across 11 functional annotations [18]. These comprised DNase I hypersensitivity sites (DHS), fetal DHS, DNase I digital genomic footprinting (DGF) regions, several histone modifications (H3K4me1, H3K4me3, H3K9ac, H3K27ac), introns, promoters, transcription factor binding sites (TFBS), and transcribed regions. Statistical significance was assessed using Bonferroni correction, applying a threshold of *p* < 0.05/(6 × 11).

#### 2.2.4. Cross-Trait Meta-Analysis

To identify specific genetic variants shared between mood swings and measures of lung function or respiratory diseases, we applied the S_Het_ statistic within the Cross-Phenotype Association (CPASSOC) framework [19]. This approach, which took the sample size into consideration, conducted a fixed effect model for meta-analysis to gain association statistics across different traits. Shared signals were identified as loci that reached suggestive significance in the single-trait GWAS (*P_single-trait_* < 1 × 10^−5^) and exhibited genome-wide significance in the combined analysis (*P_CPASSOC_* < 5 × 10^−8^). A shared SNP was considered novel if it (i) failed to reach the genome-wide significance threshold in the single-trait GWAS (5 × 10^−8^ < *P_single-trait_* < 1 × 10^−5^) and (ii) was not in LD (r^2^ < 0.2) with any previously identified genome-wide significant SNPs. For functional annotation, we further mapped the nearest genes to these shared SNPs using the Ensembl Variant Effect Predictor (VEP) [20].

#### 2.2.5. Colocalization Analysis

Next, we assessed colocalization of the shared loci at the same causal variant by applying the Coloc package (v5.2.3) [21]. This tool calculates the probability of different causal relationships between two traits within a genomic region using Approximate Bayes Factors. Specifically, it considers five possible scenarios: H0 (no causal variant), H1 or H2 (a causal variant for one trait only), H3 (independent causal variants for both traits), or H4 (a shared causal variant affecting both traits). A locus would be regarded as having colocalized if the probability of a shared causal variant (PPH4) exceeded 0.5 within 500 kb of the lead SNP as shared variants [22].

#### 2.2.6. Transcriptome-Wide Association Study (TWAS)

To uncover shared mechanisms at the gene-tissue level that might implicate common biological pathways or etiologies, we performed a transcriptome-wide association study (TWAS) with FUSION [23]. This approach integrated pre-computed expression quantitative trait loci (eQTL) weights from GTEx (v8) across 49 tissues with GWAS summary statistics for each trait [24]. Analyses were conducted separately for each tissue-trait pair. We applied a false discovery rate (FDR) correction to account for multiple testing and define significant expression-trait associations. Furthermore, for genomic regions harboring multiple associated genes, we performed joint and conditional analysis to identify independent gene-tissue signals [25]. To identify shared gene-tissue pairs, we intersected the results across different traits.

#### 2.2.7. Mendelian Randomization Analysis

To look into the possibly existing causal relationships between mood swings and lung function, as well as respiratory diseases, we performed a two-sample MR analysis. Instrumental variables (IVs) that genetically represent mood swings were carefully designated by clumping genome-wide significant variants (*p* < 5 × 10^−8^), where a stringent criterion was chosen (r^2^ = 0.001 within a 10 Mb window).

The primary causal estimates were obtained through inverse-variance weighted (IVW), complemented by weighted-median, MR-Egger regression, and MR-PRESSO methods. To ensure the robustness of our MR models, we conducted several sensitivity analyses: excluding palindromic IVs, removing pleiotropic SNPs associated with potential confounders with the GWAS Catalog (https://www.ebi.ac.uk/gwas/, accessed by 10 September 2024), performing leave-one-out analysis, and applying the MR-PRESSO method. We also used Steiger filtering to exclude SNPs that explained more variance in the outcome than in the exposure [26]. Additionally, we applied multivariable MR [27] to adjust for confounding factors, such as body mass index (BMI) [28], smoking initiation [29], drinks per week [29], physical activity [30], and sleep duration [31]. Specifically, a two-step MR analysis was used to investigate BMI as a potential mediator between mood swings and lung function and respiratory diseases. To address potential sample overlap, we conducted a supplementary analysis by excluding UK Biobank participants from the lung function GWAS.

All MR analyses were carried out utilizing the following R packages: “TwoSampleMR” (v0.5.6), “MVMR” (v0.3), and “MRPRESSO” (v1.0) in R software (v4.3.2).

## 3. Results

### 3.1. Global Genetic Correlation

As our results illustrated, mood swings exhibited significant genetic correlations with multiple lung function parameters and respiratory diseases (Table 1). To specify, mood swings exhibited a significant negative genetic correlation with three lung function parameters: FEV1 (*r_g_* = −0.09, *p* = 3.00 × 10^−5^), FVC (*r_g_* = −0.09, *p* = 6.69 × 10^−5^), and PEF (*r_g_* = −0.09, *p* = 2.89 × 10^−5^). For the respiratory diseases serving as the outcome, mood swings were demonstrated to be positively correlated with both asthma (*r_g_* = 0.33, *p* = 2.34 × 10^−18^) and COPD (*r_g_* = 0.28, *p* = 2.77 × 10^−18^). After constraining the intercepts of genetic covariance values, it turned out that the findings also remained robust, adding to the significance of the discovered genetic correlations that existed between mood swings and lung function as well as respiratory diseases.

### 3.2. Local Genetic Correlation

**Figure 2:**
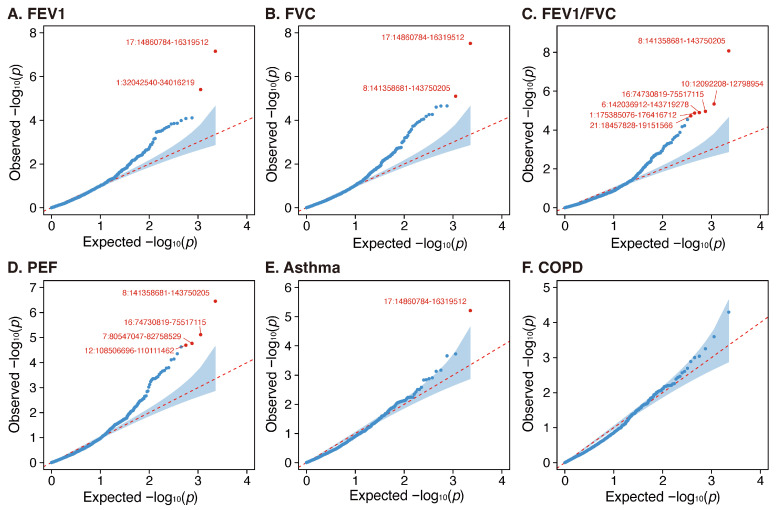
Local genetic correlation of mood swings with lung function (FEV1, FVC, FEV1/FVC ratio, and PEF), asthma, and COPD. FEV1, forced expired volume in 1 s; FVC, forced vital capacity; PEF, peak expiratory flow; COPD, chronic obstructive pulmonary diseases.

By dividing the whole genome into 2353 genomic regions, ten local genetic regions (FEV1: chr17p12-p11.2 and chr1p35.1; FVC, chr17p12-p11.2 and chr8q24.3; FEV1/FVC: chr8q24.3, chr10p13, chr16q23.1, chr6q24.1-q24.2, chr21q21.1, and chr1q25.1; PEF: chr8q24.3, chr16q23.1, chr7q21.11, and chr12q23.3-q24.11; asthma: chr17p12-17p11.2) were identified as significant regions for lung function and asthma (Figure 2A–F). Notably, the region 17:14860784-16319512 on chr17p12-p11.2, which includes the gene *PIGL* associated with both lung function and personality disorders, was found significant in three out of six analyses. Similarly, region 16:74730819-75517115 on chr16q23.1, harboring the gene *CFDP1* associated with lung function and COPD, was significant in two of six analyses.

### 3.3. Partitioned LDSC Correlation

**Figure 3:**
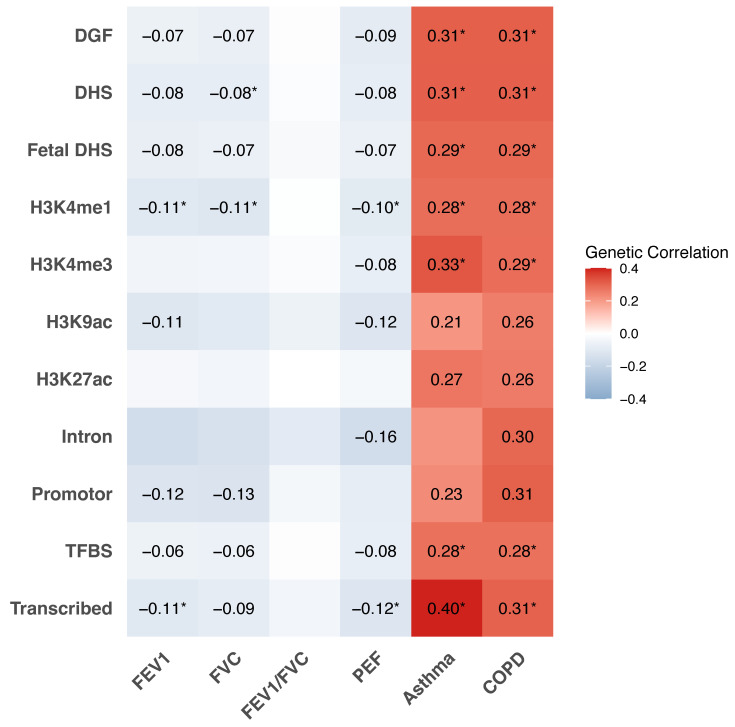
Partitioned genetic correlations of mood swings with lung function (FEV1, FVC, FEV1/FVC, and PEF), asthma, and COPD according to 11 functional categories. Numbers represent the genetic correlation at nominal significance level (*p* < 0.05); * represent significant genetic correlation after controlling for multiple testing (*p* < 0.05/6×11). FEV1, forced expired volume in 1 s; FVC, forced vital capacity; PEF, peak expiratory flow; COPD, chronic obstructive pulmonary diseases.

The results of partitioned LDSC analysis largely confirmed the global genetic correlations, revealing significant associations between mood swings with lung function and respiratory diseases in multiple functional regions (Figure 3). Significant partitioned genetic correlations were observed for mood swings with FEV1, FVC, asthma, and COPD in regions such as DGF, DHS, fetal DHS, H3K4me1, promoter, TFBS, and transcribed region, with *r_g_* values ranging from −0.07 to 0.40.

### 3.4. Cross-Trait Meta-Analysis

**Figure 4:**
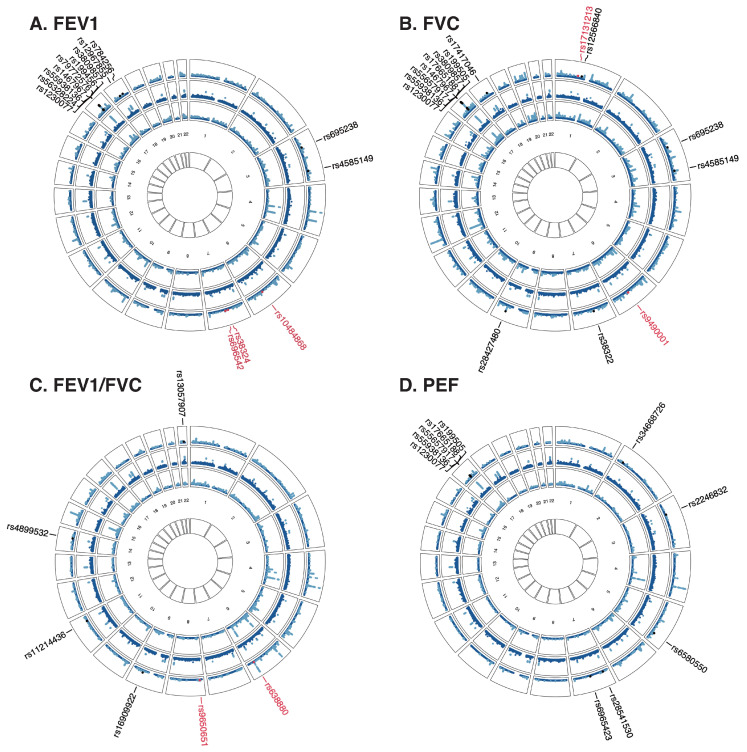
Shared genetic variants between mood swings and lung function (FEV1, FVC, FEV1/FVC, and PEF). The outermost circle shows the cross-trait meta-analysis results between mood swings and lung function; from the periphery to the center, each circle shows the GWAS results on mood swings and each lung function parameter, respectively. The light blue points represent genome-wide significant variants (*p* < 5 × 10^−8^), whereas the dark blue points represent non-genome-wide significant variants. The red points represent novel shared genetic variants, whereas the black points represent known shared genetic variants. FEV1, forced expired volume in 1 s; FVC, forced vital capacity; PEF, peak expiratory flow; COPD, chronic obstructive pulmonary diseases.

To investigate the shared genetic variants underlying the significant genetic correlations, a cross-trait meta-analysis was further conducted (Figure 4, Table S2). This analysis revealed 14, 15, 10, 6, and 10 significant independent shared loci for mood swings with FEV1, FVC, FEV1/FVC, and PEF, respectively. These SNPs were predominantly located in genomic regions chr3p21.31 (harboring *TRAIP*, *CDHR4*, *DAG1*, and *GPX1*), chr17q21.31 (harboring *CRHR1*, *MAPT*, *WNT3*, *ARL17B*, and *PLEKHM1*), and chr18q21.2 (harboring *DCC* and *TCF4*). Notably, SNPs rs79772576, rs55657917, and rs55657917 at chr17q21.31, and SNP rs16909922 at chr9q22.32 were the most significant for FEV1, FVC, PEF, and FEV1/FVC, respectively. Additionally, three novel SNPs (rs10484868 at chr6q16.1, rs38324 at chr7q11.22, and rs6965423 at chr7q21.11) were identified for FEV1, two novel SNPs (rs17131213 at chr1p22.2 and rs9490001 at chr6q16.1) for FVC, and two novel SNPs (rs638880 at chr6q24.2 and rs9650651 at chr8p23.1) for FEV1/FVC.

For asthma and COPD, one SNP rs11168048 located at 5q32 (harboring *HTR4*, *FBXO38*, and *SPINK7*) and two SNPs rs2122190 located at 5q23.1 (harboring *PRR16*) and rs6860087 located at 5q32 (harboring *HTR4*) were identified (Table S2). SNP rs2122190 was identified as a novel locus for mood swings with COPD.

### 3.5. Colocalization Analysis

Next, we performed a colocalization analysis to assess whether the genetic variants identified in the CPASSOC analysis contribute to associations across different traits. This analysis revealed multiple loci that colocalize at the same candidate SNPs, including seven shared loci for FEV1, seven for FVC, one for FEV1/FVC, four for PEF, one for asthma, and one for COPD (Table S3).

### 3.6. Transcriptome-Wide Association Study

We performed TWAS to capture genetic overlap between mood swings and lung function at the gene-tissue level. After conditional and joint analysis, we identified 16, 18, 3, and 6 genes significantly associated with mood swings and FEV1, FVC, FEV1/FVC, and PEF (Tables S4–S7), respectively. Most of these significant genes were linked to gene expression data from tissues related to the nervous, respiratory, digestive, and cardiovascular systems. Notably, a substantial proportion of significant genes showed considerable overlap with those identified in the cross-trait meta-analysis: ten for FEV1 (e.g., *CDHR4* and *GPX1* at chr3p21.31, *CRHR1* and *PLEKHM1* at chr17q21.31), ten for FVC (e.g., *IP6K1* and *RNF123* at chr3p21.31, *ARL17B* and *KANSL1* at chr17q21.31), and three for PEF (e.g., *RNF123* at chr3p21.31, *ARHGAP27* and *LRRC37A* at chr17q21.31). These genes were largely enriched in tissues including the nervous system, lung, esophagus, artery, skin, and adipose tissue.

For asthma and COPD, the conditional and joint analysis did not identify any significant independent TWAS genes. This suggests that the initial gene-tissue associations observed for these diseases were likely driven by the same core genetic variants identified in the GWAS, rather than revealing independent transcriptomic mechanisms.

### 3.7. Mendelian Randomization Analysis

**Figure 5:**
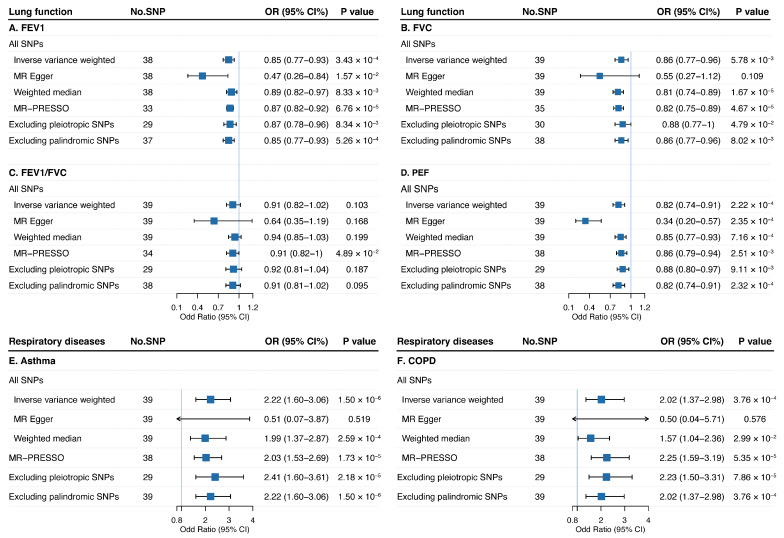
Estimates of causal effect size for genetically predicted mood swings on lung function parameters, asthma, and COPD. FEV1, forced expired volume in 1 s; FVC, forced vital capacity; PEF, peak expiratory flow; COPD, chronic obstructive pulmonary diseases.

A two-sample MR analysis was conducted utilizing 40 SNPs associated with mood swings as IVs to develop evidence for the causal effect of mood swings on lung function and respiratory diseases (Table S8). Using IVW, genetically instrumented mood swings was significantly associated with reduced lung function for FEV1 (Odd ratio [OR] = 0.85, 95% confidence interval [CI] = 0.77–0.93; *p* = 3.43 × 10^−4^), FVC (OR = 0.86, 95% CI = 0.77–0.96; *p* = 5.78 × 10^−3^), and PEF (OR = 0.82, 95% CI = 0.74–0.91; *p* = 2.22 × 10^−4^), as well as an elevated risk of asthma (OR = 2.22, 95% CI = 1.60–3.06; *p* = 1.50 × 10^−6^) and COPD (OR = 2.02, 95% CI = 1.37–2.98; *p* = 3.76 × 10^−4^) (Figure 5). These findings were further confirmed by weighted median, MR-PRESSO analysis, as well as by excluding pleiotropic or palindromic SNPs. No influential outliers were identified in the leave-one-out analysis (Figure S1). Multivariable MR analysis adjusting for potential confounders yielded comparable results and statistical significance, suggesting that the causal relationship between mood swings and lung function or respiratory diseases was largely robust to confounding factors except BMI (Figure S2). Mediation analysis suggested that BMI mediated the causal pathway from mood swings to lung function and respiratory diseases, with a significant proportion mediated for FEV1 (18.75%, 95% CI = 6.25–31.25%), FVC (26.67%, 95% CI = 13.33–40.00%), asthma (12.50%, 95% CI = 6.25–18.75%), and COPD (12.86%, 5.71–20.00%) (Table S9). Given the nonnegligible sample overlap between mood swings and lung function, we re-ran univariable and multivariable MR analysis and found consistent results after excluding subjects from the UK Biobank (Figures S3 and S4).

## 4. Discussion

To our knowledge, this study presents the first comprehensive genome-wide cross-trait analysis to investigate the shared genetic architecture between mood swings and measures of lung function as well as the risk of respiratory diseases. Our results demonstrate significant genetic correlations between mood swings and three lung function parameters (FEV1, FVC, and PEF), along with asthma and COPD. Through partitioned heritability analysis, we uncovered local genetic correlations in specific genomic regions (notably chr17p12–p11.2 and chr16q23.1) and functional annotations (including H3K4me1, DHS, and transcribed regions). Moreover, cross-trait meta-analysis identified multiple pleiotropic loci, and Mendelian randomization analysis provided evidence supporting a potential causal role of mood swings in impairing lung function and increasing the risk of respiratory diseases.

We assessed the global genetic correlations between mood swings, lung function, and respiratory diseases by applying linkage disequilibrium score regression (LDSC) with a constrained intercept [16]. This approach was selected for two key reasons: first, to address the substantial sample overlap between the GWAS of mood swings and lung function traits; second, because the observed genetic covariance intercepts significantly deviated from zero (ranging from −0.013 to 0.015), indicating potential confounding due to population stratification or other biases. The constrained intercept method is specifically designed to mitigate such issues, thereby improving the reliability of our correlation estimates, was employed for two primary reasons. Firstly, substantial sample overlap existed among the studies included in the GWAS for mood swings and lung function. Secondly, the genetic covariance intercepts deviated significantly from zero (ranging from −0.013 to 0.015), suggesting potential biases from population stratification or other confounding factors. These issues—sample overlap and non-zero intercepts—indicate possible biases that this method specifically addresses, making it appropriate for our analysis. Upon dividing the genome into 2353 genomic regions, several significant local genetic correlations were identified between mood swings with lung function parameters and asthma. For example, the region chr17p12-p11.2, significant in three of six analyses, contains *PIGL*, a gene associated with both lung function and personality disorders [12,32]. Stratified LDSC also uncovered significant genetic correlations in various annotated genomic regions, with the strongest partitioned *r_g_* found in specific non-coding regions, such as histone modification marks (such as H3K4me1 and H3K4me3) and transcribed regions.

The significant genetic correlations observed at both global and regional levels may result from pleiotropic effects of genetic components or a causal relationship between mood swings, lung function, and respiratory diseases. To investigate these possibilities, our downstream analysis identified 55 shared SNPs for mood swings and four lung function parameters, along with two respiratory diseases. Notably, several cross-trait effects we uncovered have been previously associated with nervous system development (*CRHR1* and *MAPT*) [33,34], inflammatory response (*WNT3* and *GPX1*) [35,36], and cell proliferation (*KANSL1* and *MIR2113*) [37,38], highlighting possible pathways that link mental health to lung function and respiratory diseases. In our colocalization analysis, multiple genes (such as *WNT3*, *HTR4*, *NSF*, *CELF4*, *PTCH1*, and *TRAIP*) demonstrated significant evidence of a shared causal mechanism (PPH4 > 0.5). Among these, we take two genes, *WNT3* and *HTR4*, which are associated with mood swings with lung function, asthma, and COPD.

*WNT3* is a critical gene associated with both psychotic disorders and lung diseases, influencing the WNT/β-catenin pathway vital for neural development and lung tissue maintenance [39,40]. Dysregulation of this pathway has been linked to neuroinflammation associated with psychotic disorders, like schizophrenia [41], and impaired lung function in diseases such as COPD and asthma [35,42]. *HTR4*, a member of the family of serotonin receptors, is widely expressed in multiple brain regions, such as the prefrontal cortex, amygdala, and hippocampus, where it has been associated with mood regulation, depression, and anxiety-related behaviors [43]. Peripherally, *HTR4* has been identified in smooth muscle cells and bronchial epithelial cells involved in lung development, and it is also associated with lung function, asthma, and COPD [44]. These findings highlight *HTR4* as a shared genetic factor between mental and respiratory health. However, further basic study is needed to validate these findings and fully unravel the underlying mechanisms.

Integrating GTEx tissue expression with GWAS data at the gene-tissue level, the TWAS analysis identified potential mechanisms shared between mood swings and lung function. The analysis highlighted multiple genes involved in neurological development, immune response, oxidative stress, and cell cycle control. Beyond the nervous and respiratory systems, our findings suggest that cardiovascular, digestive, and metabolic systems also share regulatory features, indicating that the underlying biology extends beyond just the brain and lungs. For example, emerging evidence links psychological disorders with CVD, with hypotheses pointing to inflammatory, hemostatic, and autonomic processes [45]. Additionally, CVD and respiratory diseases share several common risk factors [46], making early management of CVD crucial for preventing and treating respiratory conditions. Further research is needed to fully elucidate these complex interactions.

By performing a comprehensive univariable and multivariable MR analysis, a potential causal link between genetic predisposition to mood swings and compromised lung function as well as respiratory diseases was observed. These finding largely aligns with data from a nationwide health survey, which highlighted a higher risk of impaired lung function and COPD among individuals with psychotic disorders [47], and corroborate results from three cross-sectional studies that linked psychotic disorders with impaired lung function [48,49,50]. Similarly, an MR study recently reported that mood instability was related to increased risk of asthma [5]. Our study builds on these existing MR findings by addressing two crucial aspects: reducing reverse causality through Steiger filtering and controlling for confounders with the MVMR method. Furthermore, the mediation analysis indicated that BMI mediates the causal pathway between mood swings and lung function as well as respiratory diseases. Considering these genetic and epidemiological insights, our findings reveal a detrimental effect of mood swings on the risk of impaired lung function, asthma, and COPD. It also highlights the potential benefits of emotional management strategies for enhancing lung function and preventing respiratory diseases.

Several limitations should be acknowledged. First, our analyses were based on publicly available GWAS summary statistics, which precluded the ability to perform unified principal component or admixture analysis across all cohorts to fully account for fine-scale population structure within European samples. Although the original GWAS likely applied such controls, residual stratification may persist and represent a potential source of bias. Second, since this research involved individuals of European ancestry, the generalizability of these findings to other ethnic groups may be restricted. Third, the possibility of sample overlap could introduce bias in the genetic estimates, although sensitivity analyses with non-overlapping GWAS summary-level data supported the robustness of our findings. Validation with large-scale, non-overlapping datasets is still necessary. Fourth, our definition of ‘mood swings’ relied on a single self-report item from the UK Biobank. While this measure is practical for large-scale genetic studies and has been validated in previous research, it may not fully capture the clinical complexity and multidimensional nature of mood instability. Future studies would benefit from more comprehensive, multi-item, or clinically assessed phenotyping of mood swings to confirm and extend our findings. Lastly, while we identified potential causal links and shared mechanisms between mood swings and lung function, as well as asthma and COPD, further clinical validation is required to assess the clinical utility and potential interventions.

## 5. Conclusions

In conclusion, our findings advance the understanding of the phenotypic association between mood swings and lung function, asthma, and COPD. By identifying overlapping genetic components and significant instrumental variables, we reveal the genetic correlation, pleiotropic loci, and potential causal link. These insights suggest that improving emotional management may benefit lung function and reduce the risk of respiratory diseases.

## Figures and Tables

**Table 1:** Whole genome genetic correlations of mood swings with lung function, asthma, and COPD.

Trait 1	Trait 2	Unconstrained LDSC			Constrained LDSC		
		*r_g_*	*r_g__se*	*p* Value	*r_g_*	*r_g__se*	*p* Value
Mood swings	FEV1	−0.09	0.02	3.00 × 10^−5^	−0.12	0.01	6.43 × 10^−16^
	FVC	−0.09	0.02	6.69 × 10^−5^	−0.12	0.02	5.93 × 10^−14^
	FEV1/FVC	−0.02	0.02	0.256	−0.03	0.01	0.067
	PEF	−0.09	0.02	2.89 × 10^−5^	−0.11	0.02	1.41 × 10^−12^
	Asthma	0.33	0.04	2.34 × 10^−18^	0.26	0.02	3.88 × 10^−43^
	COPD	0.28	0.03	2.77 × 10^−18^	0.24	0.02	9.99 × 10^−42^

FEV1, forced expired volume in 1 s; FVC, forced vital capacity; PEF, peak expiratory flow; COPD, chronic obstructive pulmonary diseases.

## Data Availability

This paper analyzes existing, publicly available data. Summary statistics for mood swings are publicly available from https://www.ebi.ac.uk/gwas/ (Accessed on 10 June 2024). Summary statistics for lung function and respiratory diseases are publicly available from https://gwas.mrcieu.ac.uk/ (Accessed on 15 July 2024). The data for GTEx v8 multi-tissue expression are available from http://gusevlab.org/projects/fusion/ (Accessed on 21 April 2024).

## Supplementary Materials

The following supporting information can be downloaded at: https://www.mdpi.com/article/10.3390/jpm15110550/s1. Figure S1. Box plot of the leave-one-out analysis. Each SNP was systematically removed at a time, and inverse-variance weighted analysis was performed using the remaining SNPs. Figure S2. Multivariable Mendelian randomization analysis of genetically predicted mood swings on lung function and respiratory diseases. Figure S3. Univariable Mendelian randomization analysis of genetically predicted mood swings on lung function after excluding UK Biobank subjects. Figure S4. Multivariable Mendelian randomization analysis of genetically predicted mood swings on lung function after excluding UK Biobank subjects. Table S1. Details of GWAS summary data. Table S2. Results from local genetic correlation analysis conducted for mood swings and lung function and respiratory diseases. Table S3. Significant pleiotropic SNPs identified by cross-trait meta-analysis (PCPASSOC < 5 × 10^−8^ and P_single-trait_ < 1 × 10^−5^, clumping r^2^ = 0.2). Table S4. Results from colocalization analysis for each pleiotropic locus identified from CPASSOC. Table S5. Shared significant expression-trait associations between mood swings and FEV1 from TWAS after conditional and joint analysis. Table S6. Shared significant expression-trait associations between mood swings and FVC from TWAS after conditional and joint analysis. Table S7. Shared significant expression-trait associations between mood swings and FEV1/FVC from TWAS after conditional and joint analysis. Table S8. Shared significant expression-trait associations between mood swings and PEF from TWAS after conditional and joint analysis. Table S9. Details of instrumental variables selected for mood swings. Table S10. Mediation effect of BMI in the association between mood swings and lung function and respiratory diseases.

## List of Abbreviations

Abbreviation	Full Name
COPD	Chronic Obstructive Pulmonary Disease
CVD	Cardiovascular Disease
GWAS	Genome-Wide Association Study
MR	Mendelian Randomization
FEV1	Forced Expiratory Volume In 1 Second
FVC	Forced Vital Capacity
PEF	Peak Expiratory Flow
LD	Linkage Disequilibrium
LDSC	Linkage Disequilibrium Score Regression
DHS	Dnase I Hypersensitivity Site
DGF	Digital Genomic Footprinting
TFBS	Transcription Factor Binding Site
CPASSOC	Cross Phenotype Association
VEP	Variant Effect Predictor
TWAS	Transcriptome-Wide Association Study
GTEx	Genotype-Tissue Expression
FDR	False Discovery Rate
IVs	Instrumental Variables
IVW	Inverse-Variance Weighted
BMI	Body Mass Index
