# Supplementary material for: Genetic Association of Mood Swings with Lung Function and Respiratory Diseases
Source: J Pers Med. 2025 Nov 12;15(11):550. doi: 10.3390/jpm15110550 (PMC12653518; doi:10.3390/jpm15110550)
Supplement: Supplementary file 1 [file jpm-15-00550-s001.zip › Supplentary Figure.pdf]

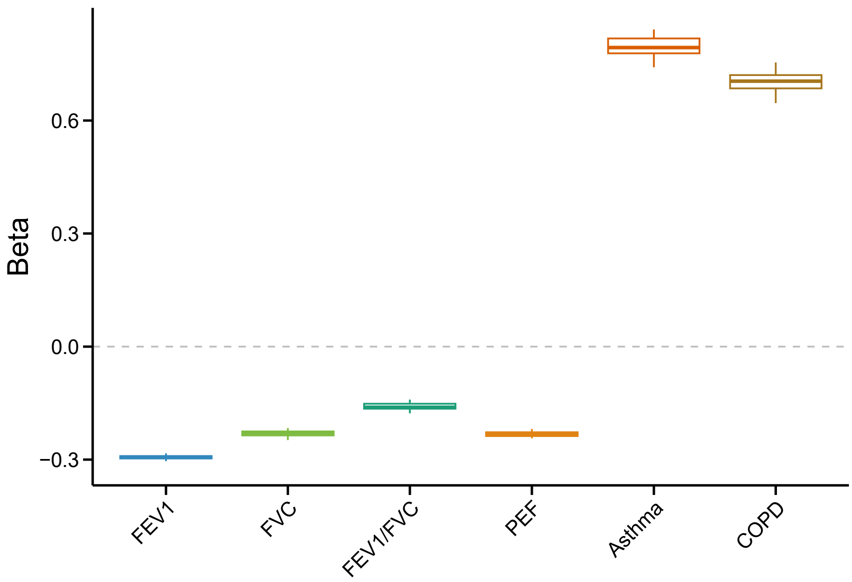

Figure S1. Box plot of the leave-one-out analysis. Each SNP was systematically removed at a time, and inverse-variance weighted analysis was performed using the remaining SNPs. Beta, effect allele beta coefficient; FEV1, forced expired volume in 1 s; FVC, forced vital capacity; PEF, peak expiratory flow; COPD, chronic obstructive pulmonary diseases.

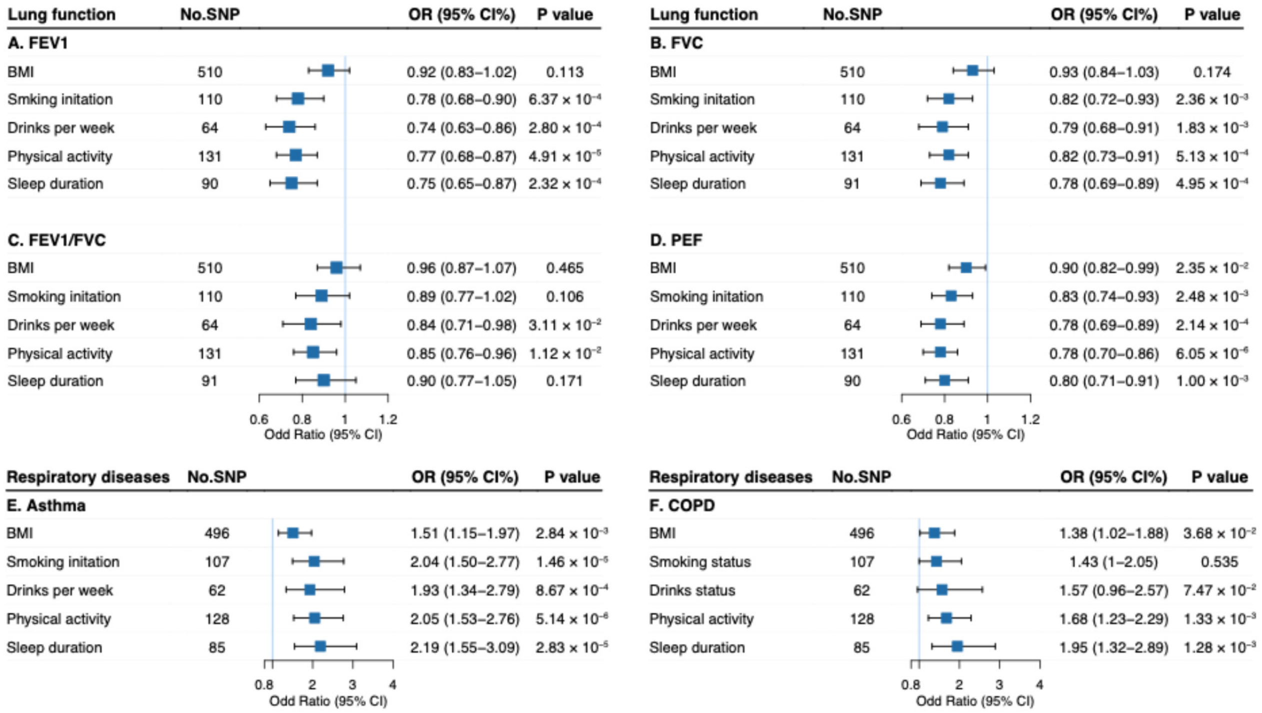

Figure S2. Multivariable Mendelian randomization analysis of genetically predicted mood swings on lung function and respiratory diseases. The estimated effect sizes were adjusted for each potential confounder separately. The y-axis indicates the genetically predicted confounder for which adjustment was made. BMI, body mass index; FEV1, forced expired volume in 1 s; FVC, forced vital capacity; PEF, peak expiratory flow; COPD, chronic obstructive pulmonary diseases.

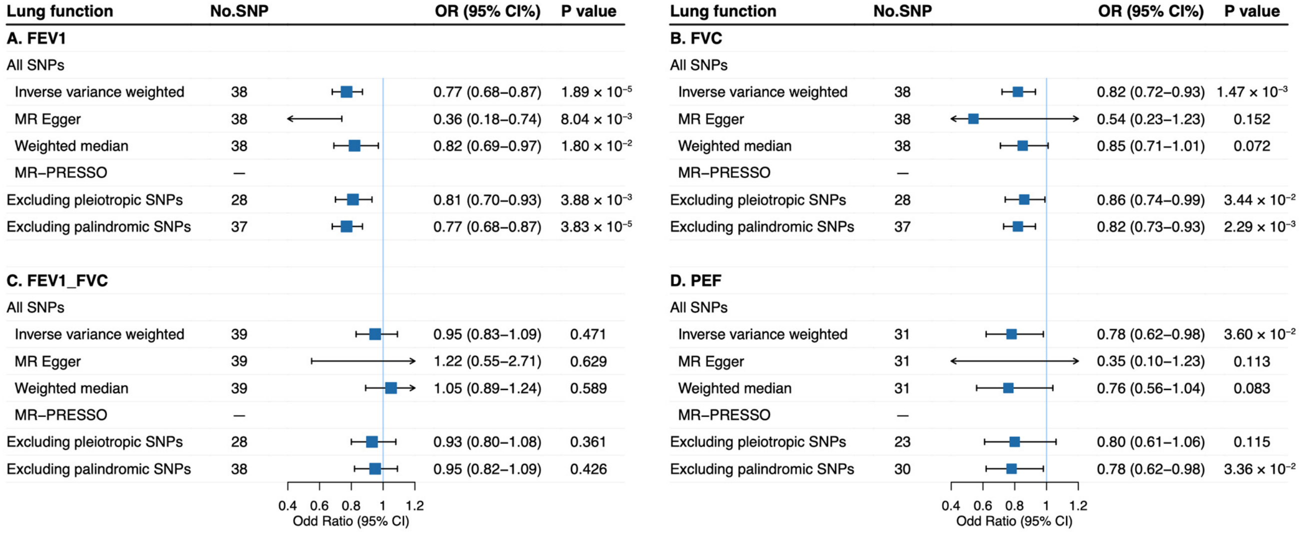

Lung function	No.SNP	OR (95% CI%)	P value
**A. FEV1**			
All SNPs			
Inverse variance weighted	38	0.77 (0.68–0.87)	1.89×10^{-5}
MR Egger	38	0.36 (0.18–0.74)	8.04×10^{-3}
Weighted median	38	0.82 (0.69–0.97)	1.80×10^{-2}
MR–PRESSO	—		
Excluding pleiotropic SNPs	28	0.81 (0.70–0.93)	3.88×10^{-3}
Excluding palindromic SNPs	37	0.77 (0.68–0.87)	3.83×10^{-5}
**C. FEV1_FVC**			
All SNPs			
Inverse variance weighted	39	0.95 (0.83–1.09)	0.471
MR Egger	39	1.22 (0.55–2.71)	0.629
Weighted median	39	1.05 (0.89–1.24)	0.589
MR–PRESSO	—		
Excluding pleiotropic SNPs	28	0.93 (0.80–1.08)	0.361
Excluding palindromic SNPs	38	0.95 (0.82–1.09)	0.426

Lung function	No.SNP	OR (95% CI%)	P value
**B. FVC**			
All SNPs			
Inverse variance weighted	38	0.82 (0.72–0.93)	1.47×10^{-3}
MR Egger	38	0.54 (0.23–1.23)	0.152
Weighted median	38	0.85 (0.71–1.01)	0.072
MR–PRESSO	—		
Excluding pleiotropic SNPs	28	0.86 (0.74–0.99)	3.44×10^{-2}
Excluding palindromic SNPs	37	0.82 (0.73–0.93)	2.29×10^{-3}
**D. PEF**			
All SNPs			
Inverse variance weighted	31	0.78 (0.62–0.98)	3.60×10^{-2}
MR Egger	31	0.35 (0.10–1.23)	0.113
Weighted median	31	0.76 (0.56–1.04)	0.083
MR–PRESSO	—		
Excluding pleiotropic SNPs	23	0.80 (0.61–1.06)	0.115
Excluding palindromic SNPs	30	0.78 (0.62–0.98)	3.36×10^{-2}

Figure S3. Univariable Mendelian randomization analysis of genetically predicted mood swings on lung function after excluding UK Biobank subjects. FEV1, forced expired volume in 1 s; FVC, forced vital capacity; PEF, peak expiratory flow.

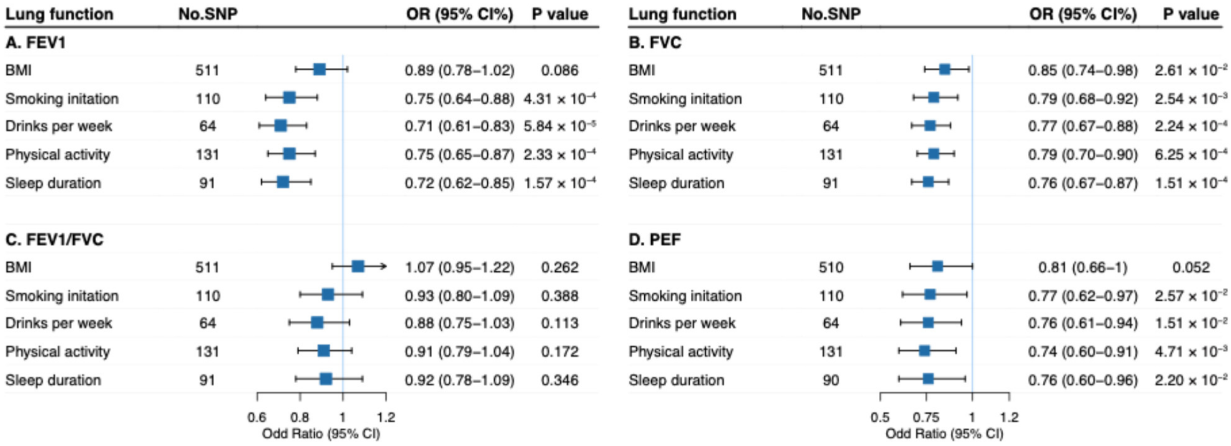

Figure S4. Multivariable Mendelian randomization analysis of genetically predicted mood swings on lung function after excluding UK Biobank subjects. The estimated effect sizes were adjusted for each potential confounder separately. The y-axis indicates the genetically predicted confounder for which adjustment was made. FEV1, forced expired volume in 1 s; FVC, forced vital capacity; PEF, peak expiratory flow.
